# Effect evaluation of a comprehensive sexuality education intervention based on socio-emotional learning among adolescents in Jakarta, Indonesia

**DOI:** 10.3389/fpubh.2023.1254717

**Published:** 2023-10-02

**Authors:** Marina Todesco, Josephine Breman, Naura Nabila Haryanto, Gerjo Kok, Karlijn Massar

**Affiliations:** ^1^Department of Work & Social Psychology, Section of Applied Social Psychology, Faculty of Psychology and Neuroscience, Maastricht University, Maastricht, Netherlands; ^2^Rutgers, Utrecht, Netherlands; ^3^Dance4Life Foundation (Closed Business), Amsterdam, Netherlands; ^4^Rutgers Indonesia, Jakarta, Indonesia

**Keywords:** sexuality education, socio-emotional learning competencies, sexual and reproductive health and rights, adolescents, evaluation

## Abstract

**Introduction:**

The field of comprehensive sexuality education (CSE) has switched from a focus on adolescents’ health to a more holistic approach, embracing the concept of sexual development with the aim of improving adolescents’ sexual well-being. The growing interest in the link between CSE and socio-emotional learning (SEL) competencies led to the development of the Journey4Life (J4L), a comprehensive sexuality education curriculum based on socio-emotional learning. In the current research, we evaluate the effectiveness of this program on perceived behavioral control, gender equality attitudes, norms, and intentions related to sexual and reproductive health and rights.

**Methods:**

To this end, we conducted a Cluster Randomized Trial among 14 senior secondary schools in Jakarta, Indonesia. A questionnaire was administered before and after implementation of the J4L program among 16–17 year old pupils; *n* = 906 completed baseline measurement, *n* = 771 completed endline measurement. With a final matched sample of *N* = 466 (65.5% girls) we conducted a mixed model repeated measures ANOVA.

**Results:**

Unfortunately, only limited and inconsistent results were found. We found that at endline, girls had stronger positive gender equality attitudes than boys; that overall attitudes towards reporting sexual violence were stronger for girls than for boys. However, contrary to our expectations, respondents had more positive attitudes toward child marriage at baseline than at endline.

**Discussion:**

Inconsistency and non-statistical significance of results limit our ability to draw firm conclusions about the effectiveness of the Journey4Life. We discuss how complex comprehensive sexuality education interventions could best be evaluated, since our study highlights the need for better conceptualization, operationalization and measurement of the interconnection between comprehensive sexuality education and socio-emotional learning.

## Introduction

Adolescence is a unique stage of life marking the transition between childhood and adulthood. It is characterized by physical, cognitive and emotional changes that trigger individuals’ desire to explore who they are and who they want to be. Living such a transitional phase, adolescents are eager for new experiences which bring new opportunities for their development but can also result in taking more risks. Societies and communities approach this life-stage trying to find a balance between the need to empower adolescents and the need to protect them (1).

Sexuality is a prominent and important area of adolescent experimentation and development. Therefore, policies, laws, regulations, programs, and guidelines in the field of Sexual and Reproductive Health and Rights (SRHR) are developed with the aim of providing adolescents with knowledge and skills to achieve sexual health *and* sexual well-being (2). However, at present, despite progress made worldwide in adolescent sexual and reproductive health, adolescents still face major challenges in pursuing sexual well-being (3).

Indonesian adolescents are no exception and constitute 17% (4) of a population of 273.5 million individuals (5). Even with enough knowledge on SRHR, Indonesian adolescents lack information on SRHR youth-friendly services (6) and still face major SRHR challenges: adolescent birth rate among 15–19 year olds is of 47.4 per 1.000 girls (7), and despite the legal age of marriage for women being raised from 16 to 19 in 2019, national regulations still allow to stipulate child marriages at local level (8, 9). At present, Indonesian adolescents live in a society compounded by contrasting progressive and conservative voices (10). On the one hand, they have the opportunity to access global knowledge around sexuality and explore their sexual subjectivity (11), whereas on the other hand, they are obliged to align their behaviors with religious values preaching sexual morality (12). In 2022 the parliament passed a law criminalizing extra-marital sex (13). This law institutionalizes values which already prescribed that adolescents, especially girls, are not supposed to have sexual activity outside marriage, except for holding hands, often under adults’ supervision (14). As a result, adolescents’ sexual development is characterized by efforts to navigate this context: relationships happen in secret, and sexuality and sexual identity are often explored online (14). However, the secrecy and lack of open communication around sexuality leads to less than optimal sexual well-being (15, 16).

One SRHR intervention aimed at fostering adolescents’ sexual health and well-being is comprehensive sexuality education (CSE). CSE is “[…] *a curriculum-based process of teaching and learning about the cognitive, emotional, physical and social aspects of sexuality. It aims to equip children and young people with knowledge, skills, attitudes and values that will empower them to: realize their health, well-being and dignity; develop respectful social and sexual relationships; consider how their choices affect their own well-being and that of others; and, understand and ensure the protection of their rights throughout their lives*” (17). Worldwide, CSE has been proven effective in improving knowledge and attitudes towards Sexual and Reproductive Health and Rights (SRHR) among adolescents, even though there is still mixed evidence around behavior change outcomes (18). Recent studies from high income countries also found that CSE increases appreciation of sexual diversity, prevents dating and intimate partner violence, and fosters healthy relationships (19). CSE curricula addressing gender and power have been found to be more successful than curricula not addressing them, specifically for reducing STIs and unintended pregnancies (20). Whether CSE influences boys and girls differently is still debated, with one study showing larger effects of a CSE curriculum on SRHR knowledge and attitudes for boys, but at the same time higher disengagement of boys from CSE content when it questions traditional masculinity (21). Le Mat (22) points out the higher interest of girls for CSE content, explaining this by the fact that many CSE programs have been ‘feminized’ and emphasize the issues that girls generally encounter.

In Indonesia CSE, called Reproductive Health Education, is mandatory for secondary schools, delivered through a collaboration between the Ministry of Health and the Ministry of Education and Culture, and is integrated within different academic subjects. The content is not comprehensive, as it focuses on abstinence, STIs and sexual violence, but leaves out topics such as contraception, consent, sexuality and other gender-sensitive concepts (23, 24).

The holistic definition of CSE reflects a recent shift in the broader field of SRHR from a risk and health focused approach, to a positive one in which sexuality is recognized as a natural component of personal development (25–27). For this reason this sex-positive approach recognizes the value of incorporating the Social–Emotional Learning (SEL) framework into CSE. SEL is a process through which individuals acquire and apply knowledge and competencies to increase awareness about themselves and others, manage their own emotions, show empathy towards others, and make responsible decisions (28, 29). SEL has been found effective in fostering young people’s well-being (30–33).

CSE based on SEL has the potential to address sexual development as part of the overall individual’s development, building competencies that can be applied in different life domains, including sexuality. Indeed, in a recent review, Cahill et al. (34) showed that both CSE and SEL share common values. Both approaches emphasize the importance of being respectful towards oneself and others, and of taking responsibilities for action. Further, both promote pedagogy based on interactive learner-centered sessions, aimed at fostering critical reflection on thoughts and behaviors, and on building skills and knowledge for making decisions consciously while keeping others in mind.

### The current research

**Figure 1:**
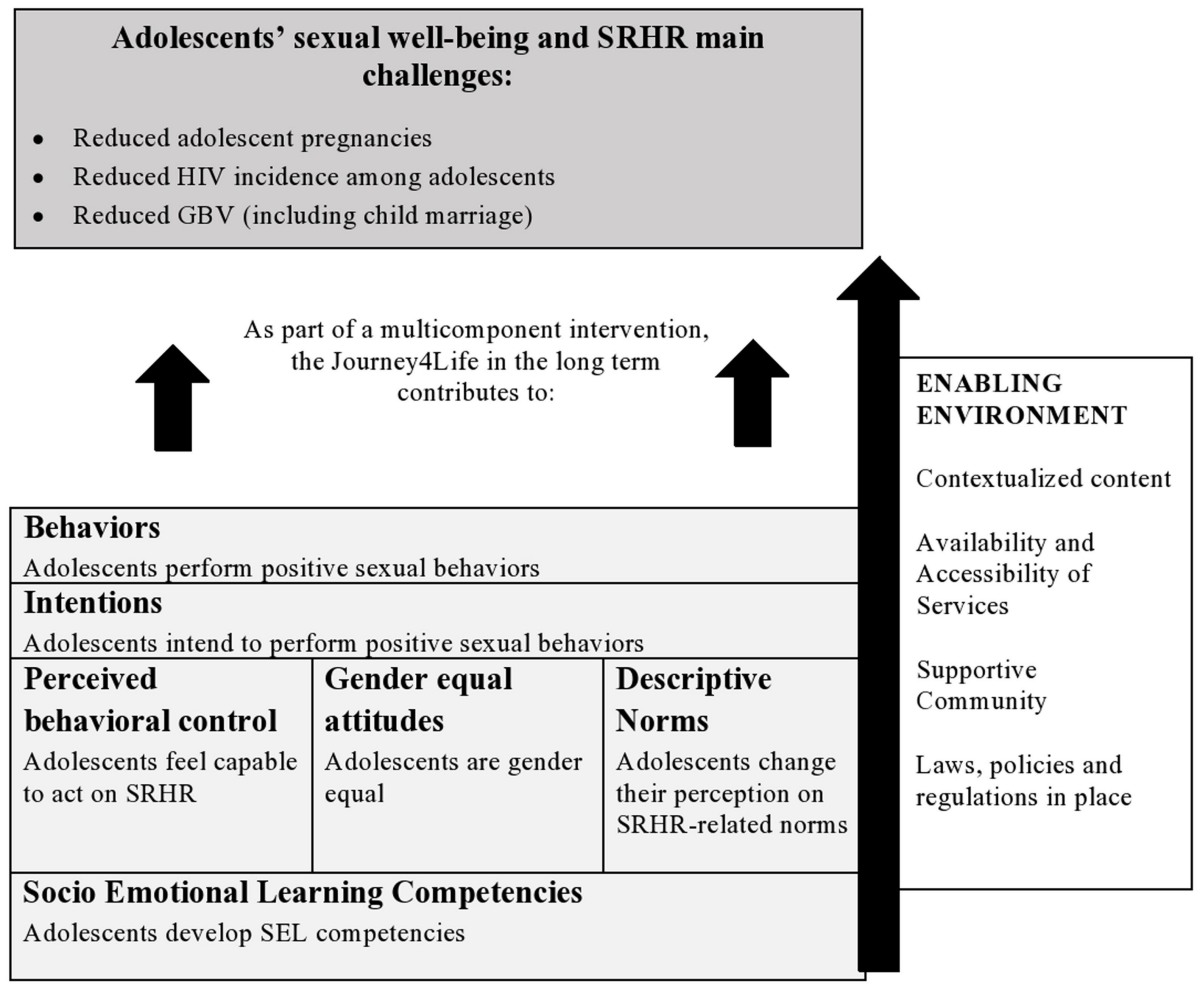
Conceptual framework of the Journey4Life.

However, although efforts to integrate skills and competencies building into CSE have been made, CSE curricula designed with a clear SEL framework in mind are scant or not known and the same ignorance applies to the their effectiveness. Our study aims to fill this gap. It explores the effectiveness of a CSE curriculum based on SEL delivered in among 16–17 year olds at 14 schools in Jakarta, Indonesia: The Journey4Life (J4L). In 2016, the Dutch organization Dance4Life developed the J4L, a peer-led CSE curriculum with a strong focus on building SEL competencies (self-awareness; self-management; social-awareness; relationship skills; decision making). Drawing from evidence of both CSE and SEL, the J4L connects them with the aim to empower young people to have positive sexual lives. The J4L is underpinned by the behavioral theory of Reasoned Action (35) highlighting that in order to behave in a sexually healthy way, young people need to form the intentions to change them (36). The J4L targets therefore the three core determinants of behavior performance – perceived behavioral control, attitudes, and norms – and fosters SEL competencies to influence these determinants, allowing young people not only to become empowered, but also to make empowered actions in relation to their sexual lives. The J4L conceptual framework (see Figure 1) shows how the SEL-based approach is largely based on the theory of Reasoned Action.

In the J4L, specific emphasis is given to gender equality. The J4L needs to be embedded in a larger multicomponent intervention to achieve its goals, specifically focusing on making sexual and reproductive health services available, accessible, and youth-friendly and implementing advocacy actions to foster community and policy support. By investigating the effectiveness of the J4L, our study aims at contributing to evidence on the impact of a CSE curriculum based on SEL, in line with a recent request made by CSE experts on the need to investigate the interconnection between SEL and CSE (37). A strong educational narrative linked to CSE can ultimately improve the dialogue with the Ministries of Education and facilitate governmental institutionalization of CSE and scale-up, especially where opposition to CSE is strong.

## Methods

### Study setting

Jakarta is a megalopolis of 4.384 Km^2^ on the northwest coast of Java island, in Indonesia. Being the sixth most populous province in Indonesia, Jakarta counts 10.55 million (38). About 544.000 adolescents are enrolled in secondary education (39). Our study targeted 16–17 year old adolescents enrolled in mixed-sex at birth classrooms of senior secondary schools in East Jakarta. The schools involved in the study were both public and private and both religious and non-religious. Approval for the materials and procedures of this study was obtained from the Ethics Review Committee of Psychology and Neuroscience (ERCPN) at Maastricht University, and checked by the local partner organization Rutgers Indonesia for context and cultural sensitivity.

### Intervention: the Journey4Life

The J4L CSE curriculum consists of 12 sessions of 90 min, over the course of 3–5 months, and is targeted at 10–24 year old youth. Sessions cover SRHR topics through a set of interactive and participatory activities. Activities are co-created together with young people from different countries using Human Centered Design approach (40) and selected by the local partner organization. Specific SRHR content can focus on one of the three main challenges experienced by young people where the CSE curriculum is implemented, which are: HIV, adolescent pregnancy; gender based violence. Peer facilitators who are selected by local partner organizations, and often have previous experience in facilitation and SRHR, deliver the J4L. Peer facilitators undergo train-of-trainers workshops in which they are trained on creative facilitation techniques and experiential learning (41). Specifically, in Jakarta, the peer facilitators completed 69 h of training on the J4L, facilitation skills, and SRHR content. This training also included a learning-by-doing approach, where trainers delivered parts of the J4L to a classroom, and a final celebration moment.

In Indonesia, the J4L was embedded in the “Get Up Speak Out for your Rights!” (GUSO) program, a multicomponent SRHR intervention integrating sexuality education, advocacy, and increase of service uptake, that took place between 2015 and 2020 in seven countries (42). The local organization Rutgers Indonesia coordinated the CSE component (J4L), which in Jakarta was implemented by local organizations: East Jakarta Red Cross, Yayasan Pelita Ilmu, and IPPA Jakarta. Guided by Dance4Life, The Country Representative and the Project Officer of Rutgers Indonesia together with six employees from the three implementing organizations and three Indonesian trainers, contextualized the content and the activities of the J4L for the Indonesian context. The decision was made to focus on decreasing gender based violence (including child marriage), and to reduce the curriculum to 10 sessions of 90 min, delivered in 3–4 months (see Supplementary Table S1 for an overview of the intervention components including goals and content).

### Recruitment and design

A Cluster Randomized Trial (CRT) was conducted in Jakarta between January and November 2019. Schools were our cluster unit. For the selection of schools we identified eight inclusion criteria: (i) schools should be located in areas with similar characteristics (in terms of socio-economic status, availability of SRHR services, and community support of SRHR); (ii) schools should be located at reasonable distance from one another to avoid spill-over effects; (iii) schools should be located in an urban area; (iv) schools should be non-sectarian; (v) schools should not focus on special education; (vi) there should be mixed classrooms (in terms of sex at birth); (vii) schools can be exposed to a national CSE curriculum, but not to other CSE curricula at the time of the intervention; and (viii) schools should not be previously exposed to the old Dance4Life interventions. However, since the research was plugged into the ongoing GUSO program, at that stage local implementing organizations did not have the option to re-negotiate the amount of schools targeted by the intervention with the local authorities. Therefore we had to be flexible in order to accommodate implementation plans already made under the GUSO program.

We employed a pre-post design, utilizing an identical self-administered questionnaire at baseline (T0) and, after three months at endline (T1) (NB: Covid-19 outbreak prevented us to access schools in order to perform the planned three-months follow up data collection). After the baseline measurement, students in the intervention schools were exposed to ten J4L sessions of 90 min (split in two sessions of 45 min), while the control schools were not exposed to any CSE curriculum until the end of the data collection (i.e., wait-list control group), when they also received the J4L. Frequency of sessions varied between schools, depending by agreements between school administrations and local implementing partners. Overall, sessions took place for a period of time of about three months, following the Indonesian academic calendar between August and November 2019.

### Included schools

Based on power calculation assuming 35 students per school, we aimed at including 30 secondary schools, to obtain ICC = 0.05 and 90% power (43). However, due to reasons provided above we were only able to include 14 schools identified by Rutgers Indonesia. Within each school two classrooms (about 70 students per school) were recruited (see also Figure 1 representing the flow diagram of participants). All of them were contacted by the local implementing organizations with the request to participate in the trial, and all 14 schools agreed to participate. Among schools selected, 10 schools were public and four private, four were religious and 10 were not, four were vocational schools while the other 10 were regular secondary schools. All 14 schools were new to the J4L, but nine of them (three in the intervention group; six in the control group) had already been exposed to a previous Dance4Life intervention.

The 14 participating schools were manually randomly assigned to the J4L CSE curriculum (*n* = 7) and to the waiting-list control condition (*n* = 7) drawing pieces of paper from a hat. Randomization was stratified to respect the proportion of schools managed by the three different local implementing organizations. This resulted in: four intervention schools and three control schools managed by East Jakarta Red Cross and IPPA Jakarta; two intervention schools and three control schools managed by Yayasan Pelita Ilmu; and one intervention and one control school managed by IPPA Jakarta. Within each school, two classrooms of 35 students (16–17 year old boys and girls) were identified by the local implementing organizations together with the school administrations. All adolescents in the two classrooms were included in the study and enrolled voluntarily.

The 14 schools identified received an information letter explaining the study and signed a “Commitment of good intentions” form. Through online and face-to-face meetings between the research team, the Country Representative of Rutgers Indonesia, and the local implementing organizations, the study aims, randomization procedure, and data collection process were explained. Once randomization was finalized, schools also signed a consent form to agree with the group they were assigned to. Young research assistants employed by the local implementing partners were trained on study objectives, methodology, and data collection. They took care of visiting the schools, explaining the study and the data collection process to the teachers and the students, managing the signature of the consent forms, and answering questions during data collection. Additionally, the questionnaire was pilot-tested among 29 senior secondary school students from Jakarta (96% girls, 16–19 years old; not attending the schools included in the study).

### Participants

**Figure 2:**
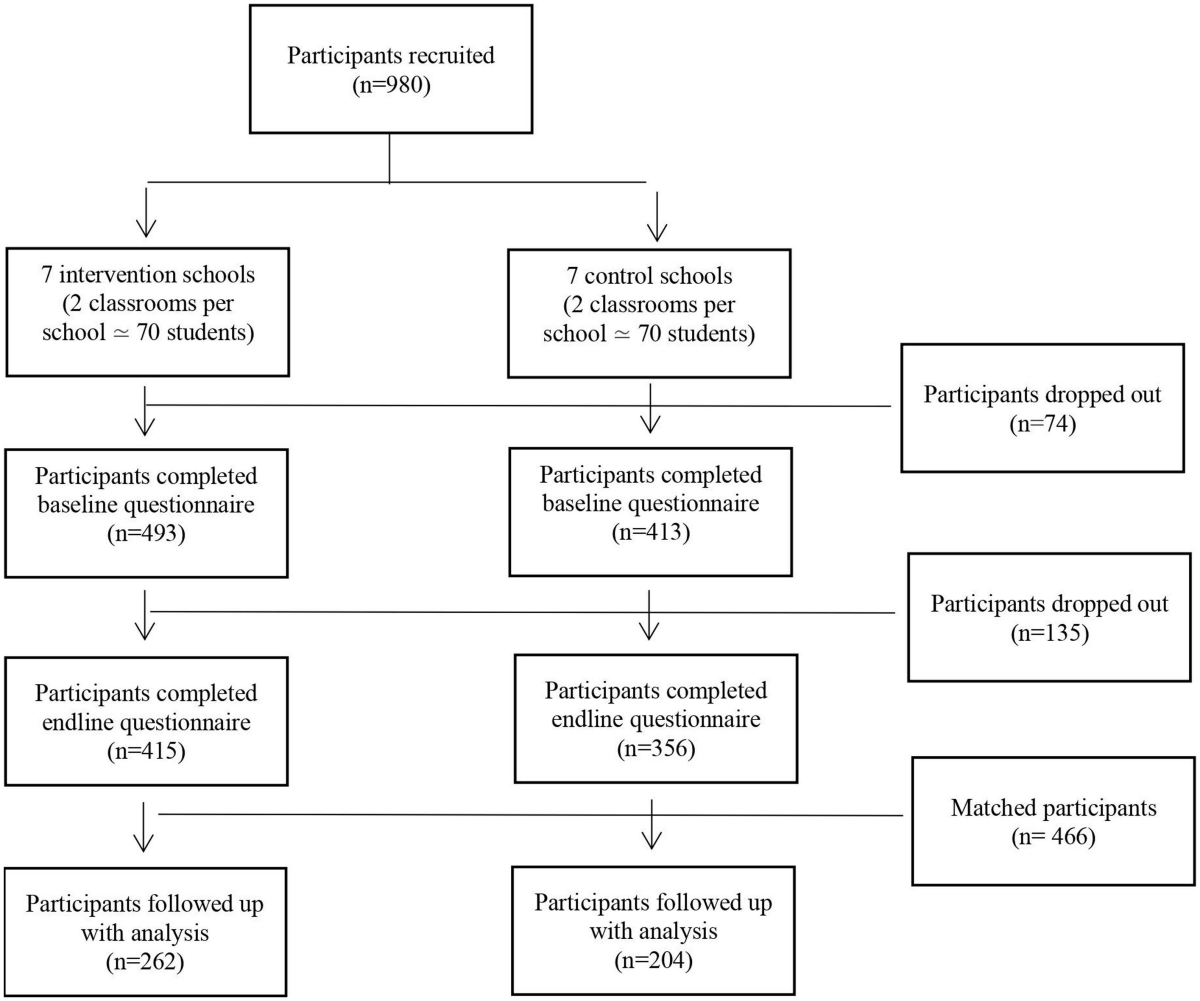
Flow diagrams of participants.

Across the 14 schools involved in the trial, a total of 980 students were targeted. As an incentive to participate in the study, Dance4Life gadgets (pens and balloons) were offered to participants. Of the 980 students reached, 906 students completed the baseline and 771 completed the endline, meaning 209 students dropped out during the course of the study (74 students before baseline, and 135 before endline). Additionally, participants that completed less than 75% of the questionnaire were excluded from the analysis, leaving 829 students at baseline and 718 at endline. Baseline and endline data were matched (using a student-generated personal code), resulting in a final sample for analysis of 466 of 16–17 year old students who completed both baseline and endline. Figure 2 shows the flow diagram of participants in the study.

### Procedure and measures

Participants received an information letter explaining their right to withdraw, anonymity, use of data, and the need to use their smartphone to fill out the online questionnaire. Then, they signed an informed consent form, and teachers’ signatures were collected. Parental consent was not mandatory due to the age (16+) of participants, but parents did receive an information letter about the study. Research assistants were present in the classroom to answer questions and collect the signed consent forms. Next, they proceeded to the survey.

Participants first completed socio-demographics: sex at birth, location (urban/rural/other), religion, name of the school, previous CSE exposure. Next, measures of perceived behavioral control about SRHR, gender equality attitudes, descriptive norms around SRHR, intentions to perform healthy sexual behaviors, and SEL competencies were administered (see Table 1 for an overview of all measures).

**Table 1:** Overview of constructs measured in the questionnaire.

Variable	Number of items	Reliability	Sample question
SEL competencies (scale)	10	α = 0.88	Imagine the following situation: A friend of yours is not sexually active but he/she has been pressured by his/her peers to have sex. Your friend ask for advice. Could you please how useful you find that advice? (5 = very useful; 1 = not useful at all)
PBC (scale)	10	α = 0.79	I feel able to either say yes or no to sexual intimacy (depending on what I want) (10 = I totally can do that, 0 = I totally can’t do that)
GEA (scale)	10	α = 0.72	A boy and a girl should decide together what type of contraception to use (4 = totally agree, 1 = totally disagree)
NORM - domestic violence	1	n.a.	Domestic violence should not be discussed outside the couple (3 = I disagree; 2 = Neither agree not disagree; 1 = I agree)
NORM - sexual violence	1	n.a.	You should not report sexual violence to the authorities (3 = I disagree; 2 = Neither agree not disagree; 1 = I agree)
NORM - pregnant at school	1	n.a.	Pregnant girls should not be allowed in school as they negatively influence peers (3 = I disagree; 2 = Neither agree not disagree; 1 = I agree)
NORM - abortion	1	n.a.	When a girl gets pregnant accidentally, abortion is never an option (3 = I disagree; 2 = Neither agree not disagree; 1 = I agree)
NORM - child marriage	1	n.a.	It is ok to get married before age 18 (3 = I disagree; 2 = Neither agree not disagree; 1 = I agree)
NORM - sexual orientation	1	n.a.	Boys are naturally attracted to girls and girls are naturally attracted to boys (3 = I disagree; 2 = Neither agree not disagree; 1 = I agree)
INTENTION - report violence	2	*r* = 0.52	Report or act against violence/bullying (done to myself or others) (4 = I tried (strong intention); 3 = I plan (middle intention); 2 = I will (weak intention); I will not = 1 (no intention))
INTENTION – express feelings	1	n.a.	Openly talk about love, feelings and emotions with my partner (4 = I tried (strong intention); 3 = I plan (middle intention); 2 = I will (weak intention); I will not = 1 (no intention))
INTENTION – seek services	1	n.a.	Visit health facilities for information or counselling on SRH (4 = I tried (strong intention); 3 = I plan (middle intention); 2 = I will (weak intention); I will not = 1 (no intention)
INTENTION - no child marriage	1	n.a.	Refuse to get married if I feel I am too young for it (4 = I tried (strong intention); 3 = I plan (middle intention); 2 = I will (weak intention); I will not = 1 (no intention))

To be able to match the baseline and endline data, we created an individual code for each participant, based on their initials, date of birth, and favorite color (e.g., *OE13.08.2003red*). The information for these codes was provided by the participants as part of the socio-demographic variables measured at both time points.

### Data analysis

Data were analyzed using IBM SPSS 25.0. A mixed model repeated measures ANOVA was performed^1^. Since previous exposure to CSE was also positively associated (*r*’s > 0.96) with the outcome variables at T0, we included this variable as a covariate. Sex assigned at birth was included in the model as factor since it could contribute to explain the results (44). Outcome variables were: perceived behavioral control (PBC), gender equality attitudes (GEA), attitudes towards harmful social norms (descriptive norms), intentions, and SEL competencies. Because of the large number of outcome variables we applied the Bonferroni correction by dividing α-level of 0.05 by the total number of outcome measures (13) resulting in a significance level of α =0.004.

## Results

### Descriptives

Among the 466 respondents who completed the questionnaire at baseline (T0) and endline (T1), 305 (65.5%) were girls and 161 (34.5%) were boys. A large majority lived in town (98.5%) and were Muslim (89.5%), followed by Christian (9.2%). In the intervention group 146 of the 262 students (55.7%) had already been exposed to some form of sexuality education, and in the control group 104 of the 204 students (51%) had already been exposed. More details about the sample’s socio-demographics are reported in Table 2.

**Table 2:** Sample’s socio-demographics.

	Total	Intervention	Control
Adolescents (16–17 year olds)	466	262	204
Sex at birth			
Girls	305	153	152
Boys	161	109	52
Location			
Town/urban	459	258	201
Village/rural	4	2	2
Other	3	2	1
Religion			
Muslim	417	232	185
Christian	43	26	17
Hindu	2	1	1
Buddhist	2	2	0
No religion (agnostic or atheist)	1	1	0
I do not want to answer	1	0	1
Previous exposure to CSE			
Yes	250	146	104
No	216	116	100

### Outcome measures

In this section, we summarize the significant findings resulting from our repeated measures ANOVAs. For a full overview of all results (i.e., interactions and main effects) we refer to Supplementary Table S2 and Table 3.

**Table 3:** Mean and *p*-value baseline and endline, intervention only.

	Mean baseline	Mean endline	*p*-value
PBC	6.71	6.75	*p* = 0.24
GEA	2.99	3.15	*p* = 0.00
SEL	3.92	3.77	*p* = 0.16
NORM – domestic violence	1.35	1.40	*p* = 0.23
NORM – sexual violence	2.73	2.71	*p* = 0.28
NORM – pregnant at school	1.84	2.00	*p* = 0.13
NORM – abortion	1.46	1.56	*p* = 0.86
NORM – child marriage	2.69	2.54	*p* = 0.002
NORM – sexual orientation	1.17	1.18	*p* = 0.54
INTENTION – express feelings	2.25	2.28	*p* = 0.75
INTENTION – seek services	2.33	2.48	*p* = 0.04
INTENTION – report violence	2.24	2.24	*p* = 0.51
INTENTION – no child marriage	2.10	2.04	*p* = 0.56

#### Perceived behavioral control

The full factorial ANOVA with condition (intervention/control), time (pre/post) and sex at birth (girl/boys) did not result in any significant results (all *p*’s > 0.22, all *F*’s < 1.51).^2^

#### Gender equality attitudes

The only significant findings for GEA were a main effect for Time, where baseline scores (*M* = 3.00, SD =0.34) were lower than endline scores (*M* = 3.13, SD =0.37). And a main effect of sex at birth, where girls showed more positive gender equality attitudes (*M* = 3.18; SD =0.35) than boys (*M* = 3.05; SD = 0.38). Also the covariate (previous exposure to CSE) was significant [*F* (1;466) =29.6, *p* = 0.000]. All other effects were not significant (all *p*’s > 0.07, all *F*’s < 3.25).

#### Socio emotional learning competencies

The full factorial ANOVA with condition (intervention/control), time (pre/post) and sex at birth (girl/boys) did not result in any significant results (all *p*’s > 0.04, all *F*’s < 1.99).

#### Descriptive norms

The only significant findings for norms were a main effect for Time on the norm “refuse child marriage”, where baseline scores (*M* = 2.65, SD = 0.594) were higher than endline scores (*M* = 2.48, SD = 0.676), and a main effect for sex at birth for the norm “reporting sexual violence”, where girls showed a more positive perception of the norm (*M* = 2.76, SD = 0.549) compared to boys (*M* = 2.57, SD = 0.723). Lastly the covariate (previous exposure to CSE) was significant for norm “reporting sexual violence” [*F* (1;466) = 9.989, *p* < 0.002] and for norm “allowing pregnant girls at school” [*F* (1;466) = 31.786, *p* = 0.000]. No other effects were significant (all *F*’s < 6.396, all *p*’s > 0.012).

#### Intentions

The only significant finding for intentions was the interaction term for Time*Condition for the “*intention to refuse child marriage*” [*F* (1;466) = 6.902, *p* < 0.01], where in the intervention group baseline scores (*M* = 2.09, SD = 0.46) were slightly higher than endline scores (*M* = 2.04, SD = 0.59); and in the control group baseline scores (*M* = 1.96, SD = 0.54) were lower than endline scores (*M* = 2.07, SD = 0.65). No other effect was significant (all *F*’s < 5.103, all *p*’s > 0.03).

## Discussion

Indonesian adolescents’ sexual wellbeing is challenged by social norms around SRHR which embrace gender stereotypes and a conservative approach to sexual development. The current study aimed to evaluate the effectiveness of an existing SEL-based comprehensive sexuality education (CSE) intervention, the Journey4Life (J4L), among 16 and 17-year-old adolescents in Jakarta, Indonesia. The J4L mainly focuses on helping adolescents build socio-emotional learning competencies they can use to healthily develop sexually. The current research is one of the few studies investigating the interconnection between CSE and SEL, and in that sense, the J4L is an innovative CSE curriculum with a strong focus on SEL. However, our findings cannot answer the question whether embedding SEL within CSE has larger effects on adolescents’ (sexual) well-being than CSE by itself.

### Key findings

Overall, unfortunately, we did not find any significant differences between intervention and control schools for any of the outcomes investigated, nor did we find consistent (and positive) changes in the outcome variables for the intervention schools between baseline and endline. Moreover, the few significant results we found do not allow us to draw firm conclusions about the effectiveness of the J4L: we found a main effect of *time* for GEA (i.e., higher scores at endline), and for the norm “refuse child marriage” (i.e., lower scores at endline). There were main effects of *sex at birth* for GEA, and for the norm “reporting sexual violence” (i.e., girls showed a more positive perception of the norm compared to boys at both time points). There was only one significant interaction, i.e., between *time* and *condition* for the “intention to refuse child marriage”.

### Findings in light of existing literature and implications

One reason for the lack of findings in the current research could be based on the Journey4Life (J4L) content. Specifically, building competencies within the sexual and relationship domains, as well as SEL competencies, can be hindered or supported by an adolescent’s environment and context, which include not only their peers, teachers and parents, but perhaps most importantly, the larger cultural norms and values system they grow up in (45, 46). Since SEL was conceptualized in the US – a highly individualized culture where an emphasis is placed on (inter)personal development and competency building – the cultural relevance of SEL competencies in the Indonesian context may be questioned. Indeed, SEL competencies might vary across cultures, and one study (47) shows that successful SEL programs can become ineffective when transferred to a different context. In addition, Williams and Deutsch (48) argue that young people’s experience of development is shaped by culture and that “competencies are often linked to the sociocultural context in which youth’s prior experiences have taken place.” Therefore, before future implementation of programs developed in the global North in educational settings in other cultural contexts, comprehensive investigation should take place regarding the SEL competencies that should be increased, young people’s perspectives on SEL, and the manner of delivery of the programs (e.g., by teachers or peer facilitators).

A second reason for lack of findings might be related to the placement of the J4L as embedded within a larger SRHR program: since the J4L was intended to be part of the GUSO multicomponent SRHR programs, it required to be supported by interventions aimed at making SRHR services available, accessible and youth-friendly, at sensitizing the community, and changing regulations, rules and laws. However these components were not all implemented (yet) in Jakarta. Kakal et al. (49) explained the non-significant results of the GUSO program in Uganda by pointing out that multicomponent interventions come with some limitations as, although planned with good intentions, they might end up being implemented in a scattered way without harmonization of components in the same geographical area, causing the program to have less impact. Therefore, future implementation efforts for SRHR multi-component programs should ensure that all stakeholders (i.e., the “owners” of the components of the intervention) are aware of, and act in accordance with, the implementation plans, before implementation actually takes place. It is highly recommended to start any multi-component intervention process by forming a planning group, in which all stakeholders – including the target population – are included and have regular meetings to streamline intervention development, implementation, and evaluation (50).

Interestingly, and related again to the central importance of the context, Kakal et al. (49) also questioned the suitability of quantitative approaches for intervention evaluation, specifically the ability of quantitative measurements to capture all variables influencing adolescents’ change process. Response bias, and especially social desirability bias, also needs to be taken into account as they might have resulted in inaccurate or false answers, in line with what respondents though us and society would expect as an answer. Some authors investigating CSE interventions (26, 51) state that RCTs, although considered the gold standard for evaluation studies, might not be the best design for CSE interventions, since they do not take into account the complexities of these interventions, where the *process* of implementation, interrelated with the context, plays a fundamental role. As alternatives to the classic RCT approach, Ivanova (52) provides a list of various methods of evaluation that might be worth considering for CSE interventions, such as the combination of process and effect evaluation, pragmatic RCTs, or realist evaluations. Since we are aware of the fact that intervention development relies on research based on the triangulation of quantitative and qualitative methods, and which includes not only an effect evaluation but also a rigorous process evaluation, as part of the current project we did conduct in-depth analysis of implementation, FGDs with peer educators and interviews with Rutgers Indonesia’s staff at endline, to supplement the quantitative effect evaluation reported here, and as part of the process evaluation.

### Methodological limitations

Reasons for not finding significant effects of the J4L intervention could be traced back to various methodological constraints placed upon the current research, some due to COVID-19 restrictions. However, the main constraint was the need to align the J4L intervention with the ongoing implementation of the GUSO program in the region. This made many schools ineligible to participate in the CRT, and resulted in 14 schools that could be enrolled in the study. Importantly, nine of these schools (six in the control group) had already implemented a previous Dance4Life CSE curriculum, but it was only during data collection that we were informed that sessions of this previous CSE curriculum had already been adapted in line with J4L, causing many of the students in our research to already have exposure to a similar CSE intervention.

One additional limitation of the current research is the mismatch between the innovative approach to CSE of the J4L, and the theoretical basis it is founded on. As mentioned in the introduction, at its core, the J4L is based on a behavior change theory (i.e Theory of Reasoned Action), with various components related to attitudes, norms, and perceived efficacy predicting intention for an actual behavior change in the domain of CSE. Our baseline and endline measurement tools were also constructed in line with this theoretical basis. Yet, the content of the J4L curriculum and the way it is delivered, are inspired by new holistic approaches to CSE, which also call for the investigation of CSE outcomes that go beyond behavior change, such as healthy sexual development, positive relationships, sexual diversity, intimate partner violence, etc. (37), and development of competencies that can help young people overcome SRHR challenges.

As a last limitation, we should mention the measurement of SEL. Since our evaluation did not aim at exploring SEL competencies *per se*, but rather at the interconnection between SEL and CSE, we moved away from traditional ways of measuring SEL (53–55). We therefore used available Positive Youth Development measurement tools (56) to construct a new SEL-competencies-as-a-function-of-SRHR scale. Even though we found a high internal reliability (Cronbach’s alpha = 0.88), its other psychometric properties still need to be determined, and interpretation of the results should therefore be cautious in the current research.

## Conclusion

The current research is an effect evaluation of the Journey4Life (J4L), an innovative comprehensive sexuality education (CSE) curriculum based on socio-emotional learning (SEL), with the aim of contributing to build new evidence on the interconnection between CSE and SEL. We assessed changes on Perceived Behavioral Control, Gender equality attitudes, descriptive norms on sexual reproductive health and rights (SRHR), intentions to perform SRHR-related behaviors, and SEL competencies. Unfortunately, most of our findings were statistically not significant, limiting the conclusions that can be drawn about the effectiveness of the J4L. We conclude that, aside from various contextual and methodological constraints that influenced the design and implementation of the current evaluation study, the ability of RCTs as golden standard to assess complex CSE interventions may be limited. Therefore, effect evaluations of CSE should always include a qualitative component and be complemented by a rigorous process evaluation that can help explain quantitative findings.

Moreover, despite the lack of results, this study allow us to raise many valuable questions on how to further explore the interconnection between comprehensive sexuality education and socio-emotional learning, highlighting the need for clarity and alignment in conceptualization of this interconnection, in how to operationalize it when designing CSE curricula and in how to measure it.

## Data availability statement

The raw data supporting the conclusions of this article will be made available by the authors, without undue reservation.

## Ethics statement

The studies involving humans were approved by Ethics Review Committee Psychology and Neuroscience (ERCPN) – Maastricht University. The studies were conducted in accordance with the local legislation and institutional requirements. Written informed consent for participation in this study was provided by the participants. Since all participants were aged, no consent was necessary from legal guardians or caretakers (see Dutch Code for Ethics in Research (2018), page 7).

## Author contributions

MT: Conceptualization, Data curation, Formal analysis, Investigation, Methodology, Project administration, Writing – original draft, Writing – review & editing. JB: Investigation, Writing – review & editing. NH: Project administration, Writing – review & editing. GK: Validation, Writing – review & editing. KM: Conceptualization, Data curation, Methodology, Supervision, Validation, Writing – review & editing.

## Footnotes

^1^At first we conducted multilevel analysis (MLMs) to take into account that students were nested within schools, but we did not find substantial variance between schools (Hessian Matrix was not positively designed); therefore, we decided to proceed with repeated measures multivariate analysis of variance. We excluded cluster-adjusted GEE or SEM because of the lack of consistent and strong intercorrelations between variables, and since our main interest it in effects within and between groups rather than in exploring relationships between outcomes measures.

^2^We checked whether previous CSE exposure had a moderator effect, but we did not find it. Therefore we treated it as a covariate.
