# Supplementary material for: Effect evaluation of a comprehensive sexuality education intervention based on socio-emotional learning among adolescents in Jakarta, Indonesia
Source: Front Public Health. 2023 Oct 2;11:1254717. doi: 10.3389/fpubh.2023.1254717 (PMC10580798; doi:10.3389/fpubh.2023.1254717)
Supplement: Supplementary file 1 [file Data_Sheet_1.PDF]

Table I: summary of the intervention components

Session title	Goals of the session	Learning objectives	Determinants	Experiential learning facilitation methods	Activities
1. Ready to take off	1. Establish group culture: agreed norms and values<br>2. Introduce participants to approach life with a growth mind-set<br>4. Inspire the participants to join the transformative Journey4Life	1. bond and connect to each other<br>2. value each other<br>3. trust the others<br>4. perceive the space as safe to disclose<br>5. think and act with creativity<br>6. see challenges as opportunities<br>7. use the language of possibility<br>9. knows what to expect from the Journey4Life	Socio emotional learning competencies	Dance, singing, reflection, journal writing	1. Peer facilitators split participants in small groups and ask them to create their own groove (rythm) to introduce themselves<br>2. Using visual materials, peer facilitators guide participants to write down a set of agreed rules and values to work effectively<br>3. Peer facilitators distribute to participants their Journal4Life and ask them to decorate and personalise them.<br>4. Peer facilitators introduce the Dance4Life vision call "A world where all young people choose to be happy" and encourage participants to sing along 5. Peer facilitators help participants to reflect on the present status of SRHR for adolescents in Indonesia

2. Your footprint	1. Introduce self-consciousness, self-compassion and empathy<br>2. Foster discussion on social norms around SRHR<br>3. Offer a reflection on the impact we have on others' lives	1. accept mistakes and learn from them<br>2. face and express emotions<br>3. communicate effectively personal stories<br>4. take time to let go<br>5. reflect on ambitions and on how these can contribute to improve others' lives	Socio emotional learning competencies, social norms	Music, mindfulness, storytelling, drawings, dance	1. Peer facilitators show clap rythmic patterns to coupled partricipants and ask them to repeat; reflection on mistakes<br>2. Peer facilitators ask participants to sit and focus on their breath and to reflect on the experience<br>3. Peer facilitators share a personal story about a SRHR challenge and guide participants into positive reflection on social norms around SRHR in Indonesia<br>4. Using drawings, peer facilitators ask participants to reflect on role models and on themselves as others' role models<br>5. Peer facilitators ask participants to dance and show them a box where they can ask anonymous questions

3. Who I am	1. Develop self-consciousness<br>2. Help understand puberty	1. recognize inner strenghts and others' strenghts<br>2. become aware of the concept of self-esteem<br>3. Understand physical, emotional and mental changes experienced in pubery<br>4. be open towards body differences	Socio emotional learning competencies	Dance, singing, drawing	1. Using singing and rythm, peer facilitators guide participant to explore themselves<br>2. Peer facilitators ask participants to draw a circle, write outside it how other see them and inside it how they see themselves; reflection on the experience<br>3. Peer facilitators divide girls and boys according to their assigned sex and ask each group to draw a silhouette of a body with visible physical, emotional and mental changes, and to reflect about changes and variety of bodies.<br>4. Peer facilitators ask participants to make a super-hero power pose and through dance mentally connect with all your people in the world

4. My dreams	1. Develop self-efficacy<br>2. Foster decision making abilities<br>3. Develop critical and creative thinking<br>4. Show SRHR services accessible in the area	1. become aware of personal dreams and ambitions and increase self-efficacy towards them<br>2. increase critical thinking and decision making skills<br>3. Indentify obstacles to achieve ambitions and share skills and tools on how to overcome them<br>4. know and access to SRHR services in the area	Socio emotional learning competencies, self-efficacy, behaviour change	Dance, singing, journal, role play	1. Using dance and singing, peer facilitators introduce the session and ask participants to summarize the previous one<br>2. Peer facilitators ask participants to imagine themeselves and their lives in 10 years, and to write their reflections on the journal<br>3. Using a simulation game, peer facilitators guide participants through a decision making process on how to overcome SRHR challenges preventing them to achieve their dreams<br>4. Peer facilitators ask participants to write in their journal opportunities and challenges to achieve their dreams<br>5. Peer facilitators bring participants to visit a youth-friendly SRHR service

5. My boundaries	1. Inform about SRHR services<br>2. Foster healthy relationships	1. increase knowledge and use of SRHR services<br>2. increase awareness on how participants relate to others and on reciprocal expectations<br>3. reflect on heathy and unhealthy intimate and sexual relationships<br>4. become aware of their boundaries and learn to respect others'	Socio emotional learning competencies, Behaviour change	Dance, drawing, writing	1. Peer facilitators guide participants to reflect on their visit to a SRHR and more in general to the availability and use of youth friendly SRHR services<br>2. Peer facilitators ask participants to draw a map of their different relationships with others and to reflect on them. They also introduces the concept of healthy sexual relationships<br>3. Peer facilitators read participants some statements about other's actions and ask them to move forward or back according to the level of acceptance of that action, and later foster discussion on differences in personal bounderies and on sexual violence<br>4. Peer facilitators ask participants to write in their journal opportunities and challenges to achieve their dreams<br>5. Peer facilitators invite participants to write a letter to someone they care for

					(or to themselves) where they make their boudaries explicit

6. I hear you	1. Develop communication skills<br>2. Foster healthy relationships	1. Learn listening skills<br>2. Learn conflict and negotiation skills<br>3. increase knowledge on STIs prevention methods<br>4. say NO to unsafe sex	Socio emotional learning competencies, behaviour change	Dance, roleplay, reflection, journal	1. Peer facilitators invite participants to reflect on what they learned halfway through the Journey<br>2. Peer facilitators pair up participants and ask each of them to listen to the other without interrupting for 3 minutes, and to reflect on the experience<br>3. Peer facilitators divide participants in 6 groups and ask each to use a different negotiation strategy to solve a condom use scenario. After the experience they guide participants in the reflection about negotiation strategies and share STIs prevention methods with participants to increase their negotiation knowledge and power<br>4. Peer facilitators invite participants to write on the journal how they would say NO to unsafe sex and negotiate for safe sex

7. Peer pressure	1. Summarize Journey4Life learnings<br>2. Help young people recognize and manage peer pressure	1. refresh SRHR knowledge<br>2. resist peer pressure	Socio emotional learning competencies, behaviour change	Dance, quiz, theater role play, journaling, singing	1.Peer facilitators engage participants in a dance competition where they have to create movements for self-expression<br>2. Peer facilitators engage participants in a quiz to test the SRHR knowledge acquired in the previous sessions<br>3. Using theatre role play, peer facilitators ask participants to perform a show based on SRHR-related peer pressure and guide them into the decision making process<br>4. Peer facilitators ask participants to write in their journal what they learned about resisting peer pressure and invite them to create a small rap about it

8. Gender equality	1. Foster gender equality	1. learn about gender norms<br>2. learn to question gender norms<br>3. learn to change gender norms	Socio emotional learning competencies, gender equal attitudes, social norms	Dance, storytelling, quiz, drawing, journaling	1. Using a story, peer facilitators guide the participants into reflection about stereotypes on masculinity and femininity<br>2. Peer facilitators ask participants to link some words to "males" or "females" and help them reflect on the difference between sex and gender.<br>3. Peer facilitators divide participants by assigned sex and ask them to write down expectations about behaviours performed by the opposite sex. Later they guide participants in the discussion about gender stereotypes and gender norms<br>4. Peer facilitators ask participants to write in their journal how they would act if they were of the opposite sex in a way that is against the gender norms<br>5. Peer facilitators activate young people to go home and do something that they think the other sex would do and to write in their

					journal about the experience

9. My rights	1. Become aware of SRH rights 2. Foster inclusivity and tolerance<br>3. Develop advocacy skills	1. learn about social norms<br>2. learn how to make positive change<br>3. learn about diversity and discrimination	Socio emotional learning competencies, social norms, behaviour change	Dance, drawing, role play	1. Inspired by the game Charade, peer facilitators assign a role (gay, sex worker, HIV positive etc...) to each participant and ask the others to act towards each other in relationt to that role. Later each of them guess its role and reflect with the others about diversity and discrimination<br>2. Peer facilitators divide participants in small groups and assign a "right" to each group which has to reflect on it and create an advocacy campaign poster<br>3. Peer facilitators invite participants to write and draw on their journal what they learned about rights during the session

10. Impacting society	1. Act to change the society	1. Reflect on the social norms in the community<br>2. Learn how to make an action plan	Socio emotional learning competencies, social norms, behaviour change	Dance, drawing, reflection	1. Peer facilitators invite small groups of participants to draw their communities, what does not work and how it can be improved and ask them to present their canvas to the others.<br>2. Using drawing, peer facilitators ask participants to reflect on how they can take action to change their communities and to team up with participant with similar ideas in order to plan an action.
11. Time to celebrate	1. Celebrate inner change and willingness to make a change in the society	1. Have fun	Socio emotional learning competencies	Dance, music, debates, speeches	1. Participants organize and coordinate the celebration event
