# Supplementary material for: Effect evaluation of a comprehensive sexuality education intervention based on socio-emotional learning among adolescents in Jakarta, Indonesia
Source: Front Public Health. 2023 Oct 2;11:1254717. doi: 10.3389/fpubh.2023.1254717 (PMC10580798; doi:10.3389/fpubh.2023.1254717)
Supplement: Supplementary file 2 [file Data_Sheet_2.PDF]

Table 4 : Overview of results

	PBC	GEA	SEL
Variable	F(1;466)	F(1;466)	F(1;466)
Previous exposure to CSE (covariate)	8.075**	29.606**	1.434
Time	1.358	26.135**	1.987
Condition	1.020	1.916	4.414*
Sex at birth	1.369	13.323**	0.626
Time*Condition	1.505	3.254	5.460*
Time*Condition*Sex at birth	0.519	0.614	0.545

*p=0.05;**p=0.01

	NORM – domestic violence	NORM – sexual violence	NORM – pregnant at school	NORM - abortion	NORM – child marriage	NORM – sexual orientation
Variable	F(1;466)	F(1;466)	F(1;466)	F(1;466)	F(1;466)	F(1;466)
Previous exposure to CSE (covariate)	0.949	9.989**	31.786**	0.763	0.728	2.847
Time	1.444	1.153	2.290	0.031	9.399**	0.362
Condition	0.320	2.810	0.948	0.989	6.396*	0.201
Sex at birth	0.018	12.748**	3.051	5.614*	1.688	2.365
Time*Condition	0.217	0.278	0.011	0.973	0.488	0.199
Time*Condition*Sex at birth	0.988	0.015	0.234	0.001	0.025	0.282

*p=0.05;**p=0.01

	INTENTION – express feelings	INTENTION – seek services	INTENTION – report violence	INTENTION – no child marriage
Variable	F(1;466)	F(1;466)	F(1;466)	F(1;466)
Previous exposure to CSE (covariate)	1.166	6.355*	2.153	1.877
Time	0.103	4.439*	0.434	0.340
Condition	0.066	0.918	0.069	0.142
Sex at birth	0.173	4.061*	2.450	5.103*
Time*Condition	0.053	1.501	4.051*	6.902**
Time*Condition*Sex at birth	1.493	1.800	2.021	4.683*

*p=0.05;**p=0.01
